# The Effect of Oil Viscosity on Droplet Generation Rate and Droplet Size in a T-Junction Microfluidic Droplet Generator

**DOI:** 10.3390/mi10120808

**Published:** 2019-11-23

**Authors:** Junyi Yao, Fan Lin, Hyun Soo Kim, Jaewon Park

**Affiliations:** 1 Department of Electrical and Electronic Engineering, Southern University of Science and Technology, Shenzhen 518055, China; 2 Korea Institute of Machinery and Materials, Daegu Research Center for Medical Devices and Rehabilitation, Daegu 42994, Korea

**Keywords:** oil viscosity, T-junction droplet generator, droplet generation rate, droplet size

## Abstract

There have been growing interests in droplet-based microfluidics due to its capability to outperform conventional biological assays by providing various advantages, such as precise handling of liquid/cell samples, fast reaction time, and extremely high-throughput analysis/screening. The droplet-based microfluidics utilizes the interaction between the interfacial tension and the fluidic shear force to break continuous fluids into uniform-sized segments within a microchannel. In this paper, the effect of different viscosities of carrier oil on water-in-oil emulsion, particularly how droplet size and droplet generation rate are affected, has been investigated using a commonly used T-junction microfluidic droplet generator design connected to a pressure-controlled pump. We have tested mineral oils with four different viscosities (5, 7, 10, and 15 cSt) to compare the droplet generation under five different flow pressure conditions (*i.e.*, water flow pressure of 30–150 mbar and oil flow pressure of 40–200 mbar). The results showed that regardless of the flow pressure levels, the droplet size decreased as the oil viscosity increased. Average size of the droplets decreased by approximately 32% when the viscosity of the oil changed from 5 to 15 cSt at the flow pressure of 30 mbar for water and 40 mbar for oil. Interestingly, a similar trend was observed in the droplet generation rate. Droplet generation rate and the oil viscosity showed high linear correlation (R^2^ = 0.9979) at the water flow pressure 30 mbar and oil flow pressure 40 mbar.

## 1. Introduction

Droplet-based microfluidics entails microdevices that produce and manipulate discrete segments of one fluid within a second immiscible carrier fluid (e.g., water-in-oil droplet emulsion) [1]. This technology generates a large number of monodisperse droplets (typically in the range of tens to hundreds of micrometers) within a short period of time [2]. Each droplet has femto-, pico-, or nano-liter aqueous volume functions as an independent bioreactor, and these droplets can be individually transported, mixed, and analyzed, where massive processing and experimentation can be carried out simultaneously [3,4]. In addition, the droplet-based microfluidics can provide encapsulation of a single cell within a droplet, which allows for high-throughput single cell screening and analysis capabilities [5,6]. The droplet-based microfluidic systems have been successfully utilized in a variety of applications, such as single cell analysis/screening [7,8,9], protein crystallization [10], polymer chain reaction (PCR) [11], drug discovery [12,13,14], and microorganism reactions [15,16] to name a few. For all of these applications, producing droplets with controlled sizes as well as at controlled generation rates are key factors to obtain consistent and robust results. 

There are three commonly used microfluidic droplet generation methods: a T-junction channel configuration, a flow-focusing configuration [17], and immiscible fluid interfusion by the orifice [18,19]. The T-junction configuration utilizes the shear force imposed by an immiscible flow fluid to generate droplets at the intersection of two perpendicularly positioned microchannels [20,21,22]. This method has been used more frequently than others due to its simple design and easy operation. In the T-junction configuration, the size as well as the generation rate of droplets are influenced by many parameters, such as dimension and surface property (hydrophilicity or hydrophobicity) of the microchannels, flow rate, use of surfactants, and viscosity of the fluids [23,24]. Among these factors, viscosity of the fluids, particularly one for the continuous phase (i.e., carrier oil), is one of the dominant parameters since the viscosity is highly related to capillary number that affects the droplet break-up [25]. In addition, the shear force provided by the continuous phase is also highly related to its viscosity. Higher viscosity helps to break and create the disperse phase more easily [26]. 

In the formation of water-in-oil (W/O) emulsion droplets, the continuous phase carrier oil commonly consists of oils or water-immiscible organic solvents, which tends to be more viscous than water [27]. Although the viscosity of the continuous phase is very important in the droplet generation, its effect has not yet been well studied. The purpose of this research is to investigate the effect of different oil viscosities, 5 cSt (4.3 mPa∙s), 7 cSt (6.0 mPa∙s), 10 cSt (8.6 mPa∙s), and 15 cSt (12.9 mPa∙s), on the droplet formation such as droplet size and generation rate for T-junction microfluidic droplet generators. 

## 2. Materials and Methods 

### 2.1. Design and Fabrication 

**Figure 1:**
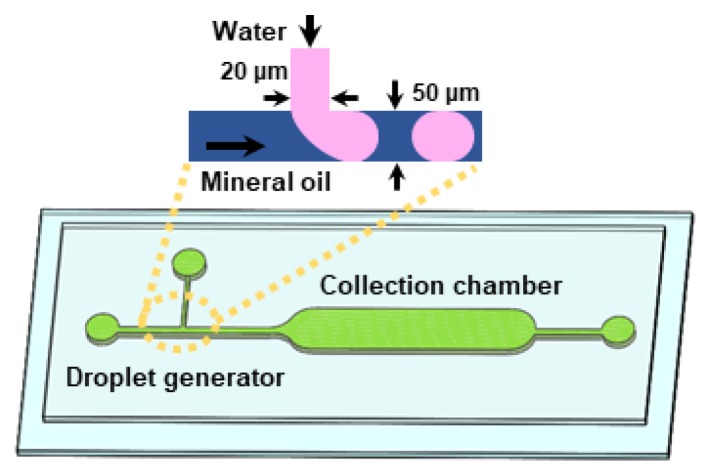
A schematic illustration of the microfluidic device that consists of a T-junction droplet generator and a droplet collection chamber. Inset shows the working principle of the T-junction droplet generation.

The microfluidic device (channel height: 33 μm) used for all experiments is composed of a T-junction droplet generator and a droplet collection chamber (Figure 1). In the T-junction droplet generator, the widths of the continuous phase (oil) and the dispersed phase (water) microchannels were designed to be 50 and 20 μm, respectively. All of the generated droplets were stored in the downstream collection chamber to characterize the droplet size as well as the generation rate.

**Figure 2:**
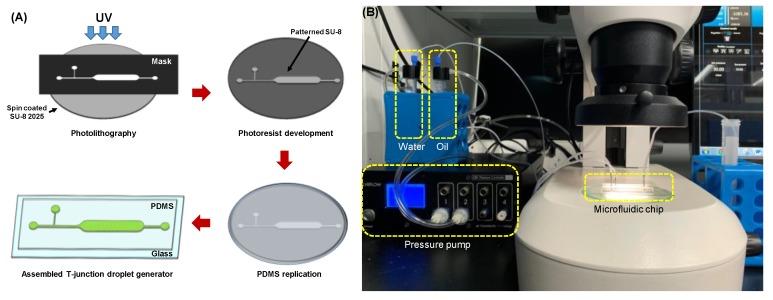
(**A**) The fabrication process of the microfluidic device. (**B**) A photograph of the experimental setup.

The microfluidic device was fabricated in poly(dimethylsiloxane) (PDMS) (Sylgard 184, Dow Corning, Midland, MI, USA) using a soft-lithography technique. First, the master mold for replicating the PDMS microfluidic device was prepared by a conventional photolithography process. A 3-inch silicon wafer was spin-coated with a negative photoresist (SU-8^TM^ 2025, Microchem Corp. Westborough, MA, USA at 2100 rpm for 30 s, then soft baked at 65 °C for 5 min, followed by additional baking at 95 °C for 15 min. The device pattern was transferred to the photoresist layer using a mask aligner (MA6, SUSS MicroTec, Garching, Germany), followed by a post-exposure bake process at 65 °C for 5 min with an additional 15 min at 95 °C. Area not exposed to UV light was removed in SU-8^TM^ developer for 2 min, and finally a SU-8^TM^ master having 33-µm-thick structures was obtained. The PDMS layer was replicated from the master by cast molding PDMS pre-polymer (10:1 mixture) for 1 h at 80 °C. Replicated PDMS layer was then treated with oxygen plasma (PDC 002, Harrick Plasma, Ithaca, NY, USA) and was permanently bonded to a glass slide (Figure 2A). After the assembly, the PDMS microchannel surfaces were coated with Aquapel^®^ (PPG Industries, Inc., Pittsburgh, PA, USA) to make them hydrophobic.

### 2.2. Droplet Generation

Instead of typical syringe pumps, a pressure-controlled pump (OB1, Elveflow, Paris, France) was used in this study to deliver both the continuous phase (i.e., oil) and the dispersed phase (i.e., water) at a constant flow. Compared to syringe pumps, pressure-controlled pumps can provide more stable and pulseless flow, handle much larger volume (up to several liters), and make it easier for the systems to be integrated with other microfluidic components as well as to build compact platforms [28]. Deionized water with red color dye and mineral oil (Zhanhong Chemical Industry, Guangzhou, China) mixed with a surfactant (Span-80, 7.5 g/L, Sigma-Aldrich, St. Louis, MI, USA) were used as the dispersed and continuous phase, respectively. Four different viscosities (5, 7, 10 and 15 cSt) of the mineral oils were tested to investigate how the different viscosities of the continuous phase affects droplet size and droplet generation rate in water-in-oil emulsion. The ratio of flow pressure between the water and the oil was set to 3:4 for all experiments conducted in this study because this ratio showed the most stable droplet generation regardless of the tested oil viscosity. Five different pressure levels tested for the droplet generation were 30:40, 60:80, 90:120, 120:160, and 150:200 mbar (P_W_:P_O_). The experimental setup is shown in Figure 2B.

### 2.3. Data Analysis

To characterize the droplet generation rate and droplet size, the droplet generation of all experiments were recorded using a stereomicroscope (NSZ608T, Jiangnan, Nanjing, China). The droplet generation rate was measured by counting the number of droplets created for each experimental condition. The droplet size was analyzed by measuring the diameters of the droplets (ImageJ, NIH, Bethesda, MD, USA) from the images captured from the recorded videos. The data shown in the manuscript are representative results from at least three independent experiments (*n* ≥ 3). 

Relative oil flow rate among different viscosity mineral oils was calculated based on Poiseuille’s Law (Equation (1)), which shows that volumetric flow rate (Q) is inversely proportional to the liquid viscosity [29]. Volumetric flow rate of 15 cSt mineral oil flown at 30 mbar was used as a reference (i.e., relative flow rate = 1), and all other calculated volumetric flow rates were normalized based on that of the reference. 

(1) Q=[1−0.63 (height)/(width)][(height)^3(width)](pressure different)12η(length)

## 3. Results and Discussion

### 3.1. Effect of the Continuous Phase Viscosity on Droplet Sizes

**Figure 3:**
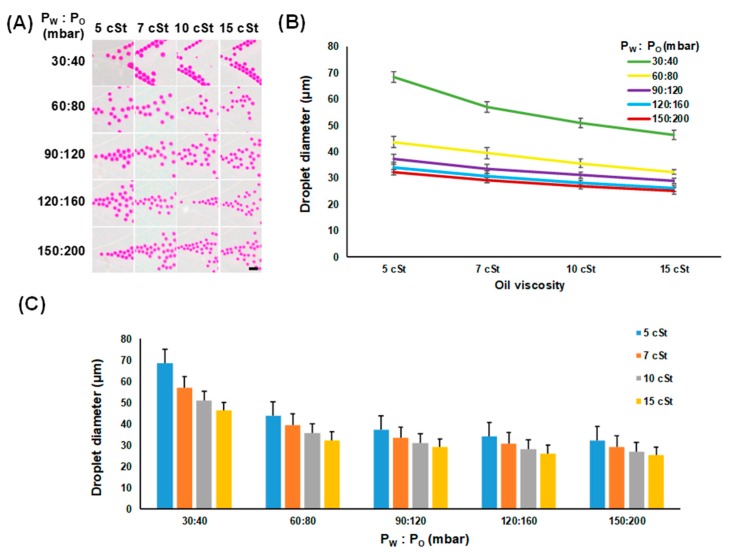
Effect of the oil viscosity on the generated droplet size. (**A**) Captured images showing the droplet formation with different sizes under various oil viscosity and flow pressure conditions (scale bars = 50 μm). (**B**,**C**) Analysis of the average droplet sizes by different viscosity and flow pressure conditions.

First, in order to examine how the oil viscosity difference affects the droplet size, the diameters of droplets created from all different oil viscosity and flow pressure conditions were measured and compared; all measurement results are summarized in Table 1. As can be seen in Figure 3A,B, the droplet size decreased as the oil viscosity increased. For instance, the average droplet size reduced from 37.1 to 33.4 (10.0% decrease), 31.0 (16.4% decrease), and 28.9 μm (22.1% decrease) as the viscosity changed from 5 to 7, 10, and 15 cSt at the flow pressure of 90:120 mbar (P_W_:P_O_), respectively. The declining trend of the droplet size displayed similar profiles over the increasing oil viscosity (10 ± 0.3% (7 cSt), 17 ± 1.1% (10 cSt), and 23 ± 2.0% (15 cSt) compared to 5 cSt) at all flow pressure conditions, except for the flow pressure of 30:40 mbar, where the average size decreased 17% (7 cSt), 25% (10 cSt), and 32% (15 cSt) against 5 cSt. 

In addition, the effect of the different flow pressure levels on the droplet sizes was analyzed. As illustrated in Figure 3C, as the flow pressure became higher, the average droplet sizes for all different oil viscosity conditions dropped. This result is consistent with a previous study where the volume of droplets decreased with an increase in the carrier fluid flow rate (i.e., the capillary number increased) [30]. The change was the most significant when the flow pressure changed from 30:40 to 60:80 mbar (P_W_:P_O_) with approximately 36%, 31%, 30%, and 30% decreases in average droplet size for 5, 7, 10, and 15 cSt, respectively.

### 3.2. Effect of the Continuous Phase Viscosity on Droplet Generation Rates

**Figure 4:**
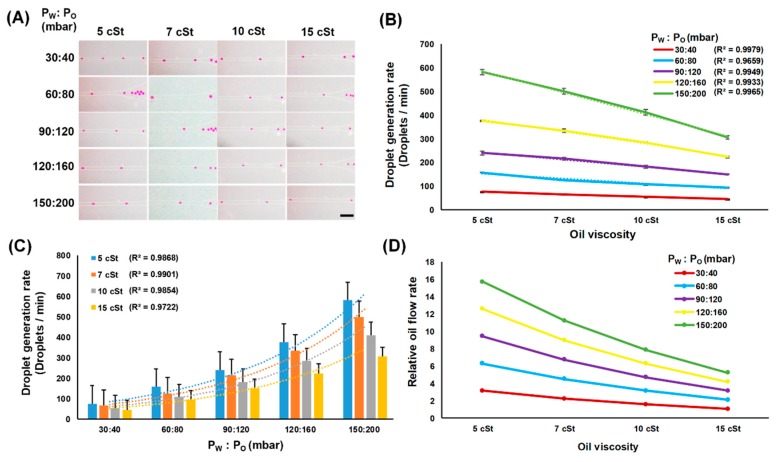
Effect of the oil viscosity on droplet generation rate. (**A**) Captured images showing different droplet generation rates under different oil viscosity and flow pressure conditions (scale bar = 100 μm). (**B**,**C**) Analysis of the droplet generation rate change by different viscosity and flow pressure levels. (**D**) Relative flow rates of the oils under all different viscosity and flow pressure conditions.

We also analyzed the effect of the oil viscosity on the droplet generation rate. As shown in Figure 4A,B, the number of droplets generated per unit time decreased as more viscous oil was used under all tested flow pressure conditions, where strong linear correlations were observed (R^2^ > 0.99 for all flow pressure conditions). For example, at a flow pressure level of 90:120 mbar (P_W_:P_O_), the droplet generation rate reduced from 239 to 215 (10% decrease), 182 (31% decrease), and 149 (38% decrease) droplets/min, when the oil viscosity changed from 5 to 7, 10, and 15 cSt, respectively (R^2^ = 0.9949, Figure 4B). Similar trends were observed from all other flow pressure levels (Table 2). This result indicates that the viscosity of the continuous phase is one of the dominant factors in the droplet generation, which affects the droplet generation rate independent of the flow pressures.

The droplet generation rate is also dependent on the flow rates of both oil and water solutions. As different flow pressure levels can change the flow rates of each solution, the effect of various pressure levels on the droplet generation rates was investigated. Larger flow pressure levels, in other words, higher fluid flow of both solutions, resulted in increase of the droplet generation rates. In fact, exponential correlations were found between the droplet generation rates and the applied flow pressures (Figure 4C). When the flow pressure levels of water and oil changed from 30:40 mbar to 150:200 mbar for 5 cSt oil, the droplet generation rates increased from 76 droplets/min to 581 droplets/min (R^2^ = 0.9868). Except for the 15 cSt oil, where the droplet generation rate increased only 6.8-fold when flow pressure changed from 30:40 mbar to 15:200 mbar (P_W_:P_O_), the droplet generation rate for all other viscosities increased approximately 7.6-fold from the same flow pressure change.

One interesting phenomenon observed from this characterization is that both the droplet size and the droplet generation rate decreased as the oil viscosity increased at a fixed flow pressure level (Table 1 and Table 2). Typically, in the droplet generation, generation rates are inversely correlated to the droplet sizes. However, in our characterization, both the droplet generation rate and the droplet size showed a positive correlation, where both values either increased or decreased simultaneously. This would be mainly due to the use of pressure-controlled pumps instead of syringe pumps for controlling both the oil and the water flow. Unlike syringe pumps, which can provide constant flow rates regardless of the carrier solution properties, the pressure-controlled pump provides constant input pressure, where resulting flow rates can be different depending on the viscosity of the carrier solution. In other words, even at the same flow pressure condition, the flow rates of oil can be different depending on its viscosity (Figure 4D). For example, at the flow pressure of 30:40 mbar (P_W_:P_O_), the relative flow rates of 10, 7, and 5 cSt were 1.5, 2.2, and 3.1 times faster, respectively, than that of 15 cSt oil. We believe this is the main reason for our results showing both the droplet generation rates and the droplet size decrease as the oil viscosity increases at a fixed flow pressure level.

## 4. Conclusions

We have investigated the effect of carrier fluid viscosity on droplet generation rate and droplet size in a T-junction microfluidic droplet generator. Mineral oils of four different viscosities (i.e., 5, 7, 10, and 15 cSt) was tested at five different flow pressure levels. The results showed that both the droplet size and the droplet generation rate decrease as higher viscosity carrier oil is used for T-junction droplet generator. For example, sizes of the droplet are often controlled by changing the flow rates or ratio between the carrier oil and water but the adjustment range is rather limited since the size of the droplet is more dependent on the microfluidic structural dimensions. However, our results show that droplet sizes can be adjusted in a broader range by just changing the oil viscosity without having to redesign and fabricate a new chip. Considering that the viscosity of the carrier fluid is an important parameter in determining the droplet generation rate as well as the droplet size and that it has not yet been well investigated how the viscosity affects the droplet generation, we believe our work can provide meaningful references for those utilizing microfluidic droplet technologies.

## Figures and Tables

**Table 1:** Average diameter of droplets with different oil viscosity and flow pressure (μm).

P_W_:P_O_ (mbar)	5 cSt	7 cSt	10 cSt	15 cSt
30:40	68.3 ± 2.0	57.0 ± 1.7	51.0 ± 1.7	46.3 ± 1.8
60:80	43.6 ± 2.1	39.5 ± 1.8	35.5 ± 1.7	32.2 ± 0.9
90:120	37.1 ± 1.8	33.4 ± 2.0	31.0 ± 1.0	28.9 ± 0.9
120:160	34.0 ± 1.9	30.7 ± 0.9	28.1 ± 1.0	26.0 ± 1.1
150:200	32.1 ± 1.0	29.1 ± 1.1	26.9 ± 1.1	25.1 ± 1.3

**Table 2:** Average droplet generation rate with different oil viscosity and flow pressure (droplets/min).

P_W_:P_O_ (mbar)	5 cSt	7 cSt	10 cSt	15 cSt
30:40	76 ± 1	66 ± 1	54 ± 1	45 ± 0
60:80	157 ± 0	126 ± 2	107 ± 3	93 ± 2
90:120	239 ± 8	215 ± 6	182 ± 6	149 ± 1
120:160	375 ± 3	334 ± 6	283 ± 3	223 ± 3
150:200	581 ± 12	499 ± 12	411 ± 12	305 ± 8

## References

[1] Seemann R., Brinkmann M., Pfohl T., Herminghaus S. (2012). Droplet based microfluidics. Rep. Prog. Phys..

[2] Rakszewska A., Tel J., Chokkalingam V., Huck W.T.S. (2014). One drop at a time: Toward droplet microfluidics as a versatile tool for single-cell analysis. NPG Asia Mater..

[3] Kim H.S., Guzman A.R., Thapa H.R., Devarenne T.P., Han A. (2016). A droplet microfluidics platform for rapid microalgal growth and oil production analysis. Biotechnol. Bioeng..

[4] Han S.I., Kim H.S., Han A. (2017). In-droplet cell concentration using dielectrophoresis. Biosens. Bioelectron..

[5] Mazutis L., Gilbert J., Ung W.L., Weitz D.A., Griffiths A.D., Heyman J.A. (2013). Single-cell analysis and sorting using droplet-based microfluidics. Nat. Protoc..

[6] Brouzes E., Medkova M., Savenelli N., Marran D., Twardowski M., Hutchison J.B., Rothberg J.M., Link D.R., Perrimon N., Samuels M.L. (2009). Droplet microfluidic technology for single-cell high-throughput screening. Proc. Natl. Acad. Sci. USA.

[7] Kim H.S., Hsu S.-C., Han S.I., Thapa H.R., Guzman A.R., Browne D.R., Tatli M., Devarenne T.P., Stern D.B., Han A. (2017). High-throughput droplet microfluidics screening platform for selecting fast-growing and high lipid-producing microalgae from a mutant library. Plant Direct.

[8] Kim H.S., Waqued S.C., Nodurft D.T., Devarenne T.P., Yakovlev V.V., Han A. (2017). Raman spectroscopy compatible PDMS droplet microfluidic culture and analysis platform towards on-chip lipidomics. Analyst.

[9] Pinho D., Muñoz-Sánchez B.N., Anes C.F., Vega E.J., Lima R. (2019). Flexible PDMS microparticles to mimic RBCs in blood particulate analogue fluids. Mech. Res. Commun..

[10] Zheng B., Tice J.D., Roach L.S., Ismagilov R.F. (2004). A droplet-based, composite PDMS/glass capillary microfluidic system for evaluating protein crystallization conditions by microbatch and vapor-diffusion methods with on-chip X-ray diffraction. Angew. Chem. Int. Edit..

[11] Markey A.L., Mohr S., Day P.J.R. (2010). High-throughput droplet PCR. Methods.

[12] Shembekar N., Chaipan C., Utharala R., Merten C.A. (2016). Droplet-based microfluidics in drug discovery, transcriptomics and high-throughput molecular genetics. Lab Chip.

[13] Munoz-Sanchez B.N., Silva S.F., Pinho D., Vega E.J., Lima R. (2016). Generation of micro-sized PDMS particles by a flow focusing technique for biomicrofluidics applications. Biomicrofluidics.

[14] Anes C.F., Pinho D., Munoz-Sanchez B.N., Vega E.J., Lima R. (2018). Shrinkage and colour in the production of micro-sized PDMS particles for microfluidic applications. J. Micromech. Microeng..

[15] Shi W., Qin J., Ye N., Lin B. (2008). Droplet-based microfluidic system for individual Caenorhabditis elegans assay. Lab Chip.

[16] Leung K., Zahn H., Leaver T., Konwar K.M., Hanson N.W., Pagé A.P., Lo C.-C., Chain P.S., Hallam S.J., Hansen C.L. (2012). A programmable droplet-based microfluidic device applied to multiparameter analysis of single microbes and microbial communities. Proc. Natl. Acad. Sci. USA.

[17] Seo M., Paquet C., Nie Z., Xu S., Kumacheva E. (2007). Microfluidic consecutive flow-focusing droplet generators. Soft Matter.

[18] Hong Y., Wang F. (2006). Flow rate effect on droplet control in a co-flowing microfluidic device. Microfluid. Nanofluid..

[19] Romero P.A., Abate A.R. (2012). Flow focusing geometry generates droplets through a plug and squeeze mechanism. Lab Chip.

[20] Gupta A., Murshed S.M.S., Kumar R. (2009). Droplet formation and stability of flows in a microfluidic T-junction. Appl. Phys. Lett..

[21] Gupta A., Kumar R. (2010). Flow regime transition at high capillary numbers in a microfluidic T-junction: Viscosity contrast and geometry effect. Phys. Fluids.

[22] He P., Barthès-Biesel D., Leclerc E. (2009). Flow of two immiscible liquids with low viscosity in Y shaped microfluidic systems: Effect of geometry. Microfluid. Nanofluid..

[23] Opalski A.S., Kaminski T.S., Garstecki P. (2019). Droplet microfluidics as a tool for the generation of granular matters and functional emulsions. KONA Powder Part. J..

[24] Teigen K.E., Song P., Lowengrub J., Voigt A. (2011). A diffuse-interface method for two-phase flows with soluble surfactants. J. Comput. Phys..

[25] Garstecki P., Fuerstman M.J., Stone H.A., Whitesides G.M. (2006). Formation of droplets and bubbles in a microfluidic T-junction—scaling and mechanism of break-up. Lab Chip.

[26] Jamalabadi M.Y.A., DaqiqShirazi M., Kosar A., Shadloo M.S. (2017). Effect of injection angle, density ratio, and viscosity on droplet formation in a microfluidic T-junction. Theor. Appl. Mech. Lett..

[27] Teh S.Y., Lin R., Hung L.-H., Lee A. (2008). Droplet microfluidics. Lab Chip.

[28] Zeng W., Li S., Wang Z. Characterization of syringe-pump-driven versus pressure-driven microfluidic flows. Proceedings of the 2015 International Conference on Fluid Power and Mechatronics.

[29] Sutera S.P., Skalak R. (1993). The history of Poiseuille’s law. Annu. Rev. Fluid Mech..

[30] Loizou K., Wong V.-L., Hewakandamby B. (2018). Examining the effect of flow rate ratio on droplet generation and regime transition in a microfluidic T-junction at constant capillary numbers. Inventions.

